# Immunomodulatory activity of IR700-labelled affibody targeting HER2

**DOI:** 10.1038/s41419-020-03077-6

**Published:** 2020-10-20

**Authors:** Justyna Mączyńska, Chiara Da Pieve, Thomas A. Burley, Florian Raes, Anant Shah, Jolanta Saczko, Kevin J. Harrington, Gabriela Kramer-Marek

**Affiliations:** 1 Division of Radiotherapy and Imaging, The Institute of Cancer Research, London, UK; 2 Department of Medical Biochemistry, Wroclaw Medical University, Wroclaw, Poland; 3 Department of Molecular and Cellular Biology, Wroclaw Medical University, Wroclaw, Poland

**Keywords:** Cancer immunotherapy, Preclinical research

## Abstract

There is an urgent need to develop therapeutic approaches that can increase the response rate to immuno-oncology agents. Photoimmunotherapy has recently been shown to generate anti-tumour immunological responses by releasing tumour-associated antigens from ablated tumour cell residues, thereby enhancing antigenicity and adjuvanticity. Here, we investigate the feasibility of a novel HER2-targeted affibody-based conjugate (Z_HER2:2395_-IR700) selectively to induce cancer cell death in vitro and in vivo. The studies in vitro confirmed the specificity of Z_HER2:2395_-IR700 binding to HER2-positive cells and its ability to produce reactive oxygen species upon light irradiation. A conjugate concentration- and light irradiation-dependent decrease in cell viability was also demonstrated. Furthermore, light-activated Z_HER2:2395_-IR700 triggered all hallmarks of immunogenic cell death, as defined by the translocation of calreticulin to the cell surface, and the secretion of ATP, HSP70/90 and HMGB1 from dying cancer cells into the medium. Irradiating a co-culture of immature dendritic cells (DCs) and cancer cells exposed to light-activated Z_HER2:2395_-IR700 enhanced DC maturation, as indicated by augmented expression of CD86 and HLA-DR. In SKOV-3 xenografts, the Z_HER2:2395_-IR700-based phototherapy delayed tumour growth and increased median overall survival. Collectively, our results strongly suggest that Z_HER2:2395_-IR700 is a promising new therapeutic conjugate that has great potential to be applicable for photoimmunotherapy-based regimens.

## Introduction

Immunotherapy with immune checkpoint inhibitors (ICPIs) has recently shown encouraging clinical benefits in cancer patients; however, the response rates are still limited, ranging between 10% and 50%^1,2^. The reasons for this are multifactorial and include a relatively immune-depleted (“cold”) microenvironment, an absence of tumour-infiltrating lymphocytes (TILs), a state of phenotypic exhaustion in those cytotoxic T lymphocytes (CTLs) that are present and the actions of potent negative regulators, such as regulatory T cells^3,4^. Nevertheless, a growing body of research has indicated that patients whose tumours are already infiltrated with T cells benefit from treatment with ICPIs^5^. Therefore, development of therapeutic strategies that trigger anti-tumour immunological responses by generating tumour-associated antigens from ablated tumour cell residues could significantly improve the efficacy of systemic immunotherapy.

It is widely recognised that conventional photodynamic therapy (PDT) and photoimmunotherapy (PIT) can induce immunogenic cell death (ICD), as defined by hallmarks such as translocation of calreticulin (CRT) to the plasma membrane, ATP secretion and release of high-mobility group box-1 (HMGB1) nuclear protein and heat shock proteins HSP70/90^6,7^. Accordingly, this can elicit uptake of antigenic components by dendritic cells (DCs), resulting in the expansion of antigen-specific CTLs^8–10^.

Both PDT and PIT are based on a photochemical reaction between light of a defined wavelength, a photosensitiser (PS) and molecular oxygen. The combination of these components causes cell death by generating cytotoxic molecules. However, in PIT the PS is conjugated to a highly specific monoclonal antibody (mAb) that can engage the selected target of interest, enabling a greater degree of tumour specificity than PDT^11,12^. Recent developments in antibody engineering technology have also led to a new wave of PIT-suitable agents that are built on a range of antibody fragments and affibodies^13–16^. Following excitation with near-infrared (NIR) light, these conjugates can cause rapid and irreversible selective disruption of membrane integrity and preferential killing of malignant cells, while sparing normal adjacent tissue^17,18^. This, in turn, can activate anti-tumour immunological responses, stimulated by the release of tumour-associated antigens^7,14,19,20^. Various PSs have been tested for PIT purposes and the silicon phthalocyanine dye, IRDye700DX (henceforth referred to as IR700) has, so far, shown the most favourable properties. This dye has excellent water solubility and a greater than five-fold higher extinction coefficient (2.1 × 10^5^ M^−1^ cm^−1^ at the maximum absorption wavelength of 689 nm) than other conventional PSs in the NIR part of the spectrum, which allows increased tissue penetration of the activating light^12^. Excitingly, an EGFR-targeting IR700-cetuximab conjugate (RM1929) is currently being investigated in a global Phase 3 clinical trial in head and neck cancer (https://clinicaltrials.gov/ct2/show/NCT03769506). Here, we evaluate the immunomodulatory activity of a novel affibody-based conjugate specifically targeting the human epidermal growth factor receptor 2 (HER2). For that purpose, we attached IR700 to a low molecular weight (~7 kDa), three-helix Z_HER2:2395_ affibody molecule that recognises HER2 with high selectivity and affinity. We chose HER2 as a target of interest because it is a major driver in the progression of epithelial neoplasms, including breast and ovarian cancers^21^. Furthermore, HER2 is involved in regulating cell growth, survival and differentiation through networked downstream signal transduction pathways, including PI3K/AKT/mTOR and Ras/Raf/MAPK^22^. HER2-positive tumours are also associated with resistance to certain types of chemotherapy, hormone therapy and ionizing radiation^23^. Consequently, numerous drugs specifically targeting the receptor, such as mAbs (*e.g*., trastuzumab, pertuzumab), antibody-drug conjugates (e.g., trastuzumab-emtansine T-DM1), and pan‐HER small molecule inhibitors (*e.g*., dacomitinib, afatinib), have been developed. Although, the majority of patients initially respond to these HER2-targeted agents, they frequently acquire resistance and, subsequently present disease progression within 8–18 months^24,25^. Therefore, there is an unmet clinical need for more effective therapeutic approaches that maximize target-cell killing in HER2-positive cancers to achieve substantial remissions. In this study, we demonstrate that Z_HER2:2395_-IR700-based phototherapy leads to HER2-specific cell death, release of danger-associated molecular patterns (DAMPs) which subsequently induce DC maturation in vitro. Moreover, the treatment in vivo results in significant tumour growth inhibition.

## Results

### In vitro cellular uptake and internalization of Z_HER2:2395_-IR700

**Fig. 1:**
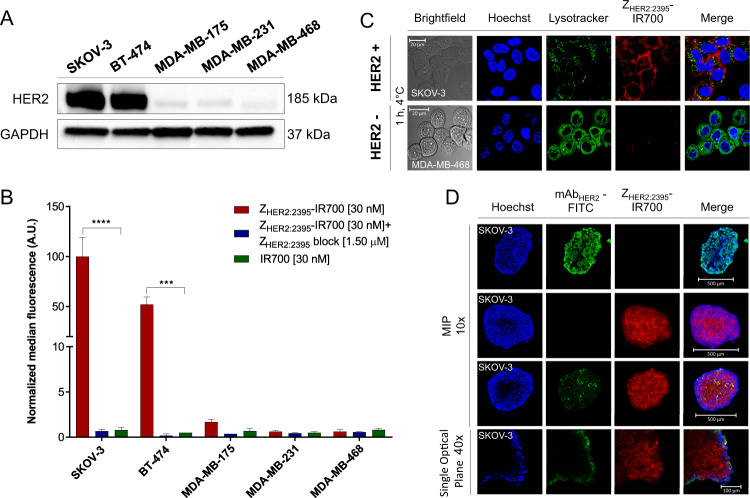
Expression of HER2 in tumour cell lines and specificity of the ZHER2:2395–IR700 binding. **A** HER2 expression levels detected by Western blot in a panel of cancer cell lines. **B** Z_HER2:2395_–IR700 (30 nM) binding (1 h, 4 °C) assessed by flow cytometry with or without blocking with 50-fold excess of unlabelled Z_HER2:2395_, in comparison to IR700 alone (30 nM). Data are presented as mean ± SEM (*n* = 3). Statistical difference in comparison to the Z_HER2:2395_–IR700 group was determined using ANOVA with Dunnett’s post hoc test. ****p ≤ 0.0001, ***p ≤ 0.001. **C** Z_HER2:2395_–IR700 (1 µM, red) cell binding (1 h, 4 °C) assessed by confocal microscopy using SKOV-3_HER2+ve_ and MDA-MB-468_HER2−ve_ cells. Hoechst^®^33342 (blue) and LysoTracker™Green DND-26 (green) were used for counterstaining. **D** Maximum intensity projections (MIPs) of SKOV-3 spheroids incubated with Z_HER2:2395_–IR700 (1 µM, red) or with anti-HER2-FITC antibody (1 µM, green) for 6 h at 37 °C. Hoechst^®^33342 (blue) was used as a counterstain.

Maleimide-functionalized IR700 was attached to a unique C-terminal cysteine on the HER2-specific affibody molecule Z_HER2:2395_. The conjugate labelling characteristics are shown in Fig. S1A, B. The binding specificity of Z_HER2:2395_-IR700 was evaluated using a panel of breast and ovarian cancer cell lines expressing different HER2 levels as indicated by the Western blot (Fig. 1A). The specific and receptor-dependent targeting of the conjugate correlated with the receptor expression level as measured by flow cytometry (Fig. 1B and Fig. S2A). The fluorescence signal was significantly reduced in the presence of 50-fold excess of non-labelled Z_HER2:2395_, confirming the conjugate binding specificity. The receptor binding affinity of Z_HER2:2395_-IR700 was assessed by flow cytometry using SKOV-3 cells (HER2-positive). The conjugate showed a high affinity for HER2 with a *K*_D_ = 7.5 ± 0.9 nM (Fig. S1C). Consistent with our prior results, cells incubated with IR700 alone demonstrated a very low uptake of the dye (Fig. 1B)^14^. Highly specific cell binding and uptake of Z_HER2:2395_-IR700 was also captured by confocal microscopy. Intense membrane fluorescence was visualised in SKOV-3 cells (HER2-positive 1 h post-incubation with the conjugate, whereas no membrane-associated fluorescence was captured in MDA-MB-468 cells (HER2-negative) (Fig. 1C). Furthermore, incubating SKOV-3 cells with the conjugate for 1, 4, or 6 h (37 °C) allowed acquisition of a distinct, cytoplasmic fluorescence signal that prominently increased over time (Fig. S2B). In addition, to investigate the penetration of the affibody conjugate, SKOV-3 spheroids were incubated with the same concentration of either Z_HER2:2395_-IR700 or mAb_HER2_-FITC for 6 h (37 °C). The spheroids treated with the affibody-based conjugate exhibited significantly higher intracellular fluorescence intensity and more homogeneous fluorescence distribution compared to spheroids incubated with mAb_HER2_-FITC, showing at the same time also greater penetration (Fig. 1D). The H&E staining of spheroid sections (~250 μm in diameter) confirmed a lack of necrotic cores and the presence of actively proliferating (Ki67-positive) HER2-positive cells within the 3D structures (Fig. S2C).

### Receptor-mediated cell death induced by Z_HER2:2395_-IR700 mediated-treatment

**Fig. 2:**
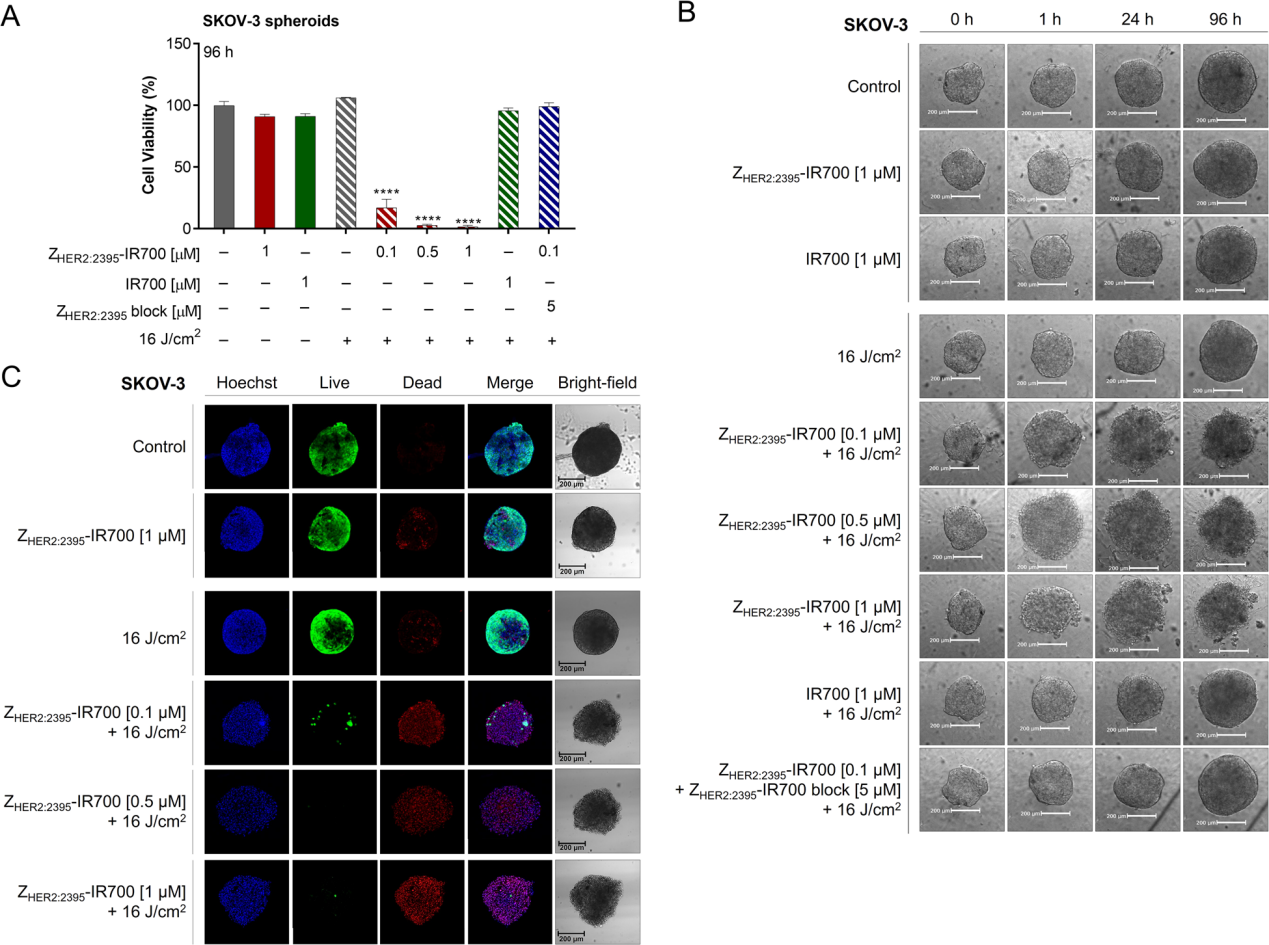
Spheroid destruction following ZHER2:2395–IR700 conjugate-based phototherapy. **A** Dose-dependent decrease in SKOV-3 spheroid viability as assessed by the CellTiter-Glo^®^ 3D luminescent cell viability assay 96 h post-treatment, following 6 h incubation with the Z_HER2:2395_–IR700 (0.1–1 µM) with or without blocking with 50-fold excess of unlabelled Z_HER2:2395_ or IR700 (1 µM) and irradiation with 16 J/cm^2^ NIR light dose. Data are presented as mean ± SEM (*n* = 3). Statistical significance in comparison to the control (untreated) group was determined using ANOVA with Dunnett post hoc test. ****p ≤ 0.0001. **B** Confocal MIP images illustrating live/dead staining assay of the SKOV-3 spheroids conducted 96 h post-phototherapy. Live—calcein AM fluorescence (live cells, green); Dead—signal from ethidium homodimer-1 bound to DNA (dead cells, red). Hoechst^®^33342 (blue) used as a counterstain of nuclei. **C** Changes in the morphology of SKOV-3 spheroids captured via Celigo® cytometer pre- and 1, 24, 96 h post-treatment.

Next, we assessed the response to Z_HER2:2395_-IR700-mediated phototherapy in vitro using SKOV-3 2D mono-layer cultures and 3D cell spheres incubated either with IR700 (1 μM) or Z_HER2:2395_-IR700 (0.01–1 μM) for 6 h (due to increased conjugate accumulation; Fig. S2B). Based on our previous work with EGFR-targeting conjugate, samples were irradiated with a light-dose of 16 J/cm^2^ to activate the photosensitiser^14^. A significant decrease (~85–97%) in cell viability within the 3D SKOV-3 spheres post-treatment was detected at 96 h and the percentage of dying cells was influenced by the concentration of Z_HER2:2395_-IR700 (Fig. 2A). In contrast, no toxicity was observed when spheroids were exposed to either Z_HER2:2395_-IR700, IR700 or light irradiation alone. Furthermore, the majority of cells were viable when the spheroids were subjected to IR700-based phototherapy or pre-incubated with 50-fold excess of non-labelled affibody molecules prior to Z_HER2:2395_-IR700 treatment, which confirmed receptor-mediated cell phototoxicity. In 2D HER2-positive monolayer cultures (BT474, SKOV-3), we also observed a significant decrease in the luminescence signal in a conjugate concentration-dependent manner 24 h post-treatment (Fig. S3A, B). Conversely, the same treatment regimen had no effect on HER2-negative MDA-MB-468 cells (Fig. S3C). Irradiation of cells incubated with IR700 dye alone resulted in considerable (~11–44%) loss in cell viability regardless of the HER2 expression level (Fig. S3A–C). In addition, there was no decrease in cell viability when the Z_HER2:2395_-IR700, IR700, or NIR light were used alone in either of the cell lines. Moreover, we observed a substantial generation of reactive oxygen species (ROS) post-Z_HER2:2395_-IR700 irradiation (Fig. S3D). Importantly, cell viability was partially rescued by pre-treatment with a radical scavenger (N-acetylcysteine, NAC) or apoptosis inhibitor (pan-caspase Z-VAD-FMK), confirming a role for oxidative stress-induced cell death in response to Z_HER2:2395_-IR700- phototherapy (Fig. S3E). Finally, in order to depict cell death morphological changes induced by Z_HER2:2395_-IR700-phototherapy, SKOV-3 spheroids were imaged 1, 24, 96 h post-irradiation. As demonstrated on the bright-field images (Fig. 2B), the treatment did not result in spheroid growth inhibition. However, we observed cellular swelling, increased membrane disruption and cell displacement from the dense sphere core, which formed a loose spheroid periphery as early as 1 h post-irradiation. In order to identify both viable and necrotic cells within the 3D spheres, live/dead fluorescence staining was additionally performed 96 h post-light exposure. The resulting maximum intensity projections (MIP) of the entire image z-stacks indicated an affibody concentration-dependent decrease in the viability of cells 96 h post-treatment. Incorporation of EthD‐1 (dead cells) confirmed significant cell damage leading to acute necrosis (Fig. 2C). In contrast, there was only negligible cell death in spheres treated with the conjugate or irradiated with light alone.

### Z_HER2:2395_-IR700-based treatment triggers the release of DAMPs in vitro

As PIT induces ICD, a mode of death where dying cancer cells release DAMPs, we next analysed major endogenous danger molecules: CRT, ATP, HSP70/90, and HMGB1.

**Fig. 3:**
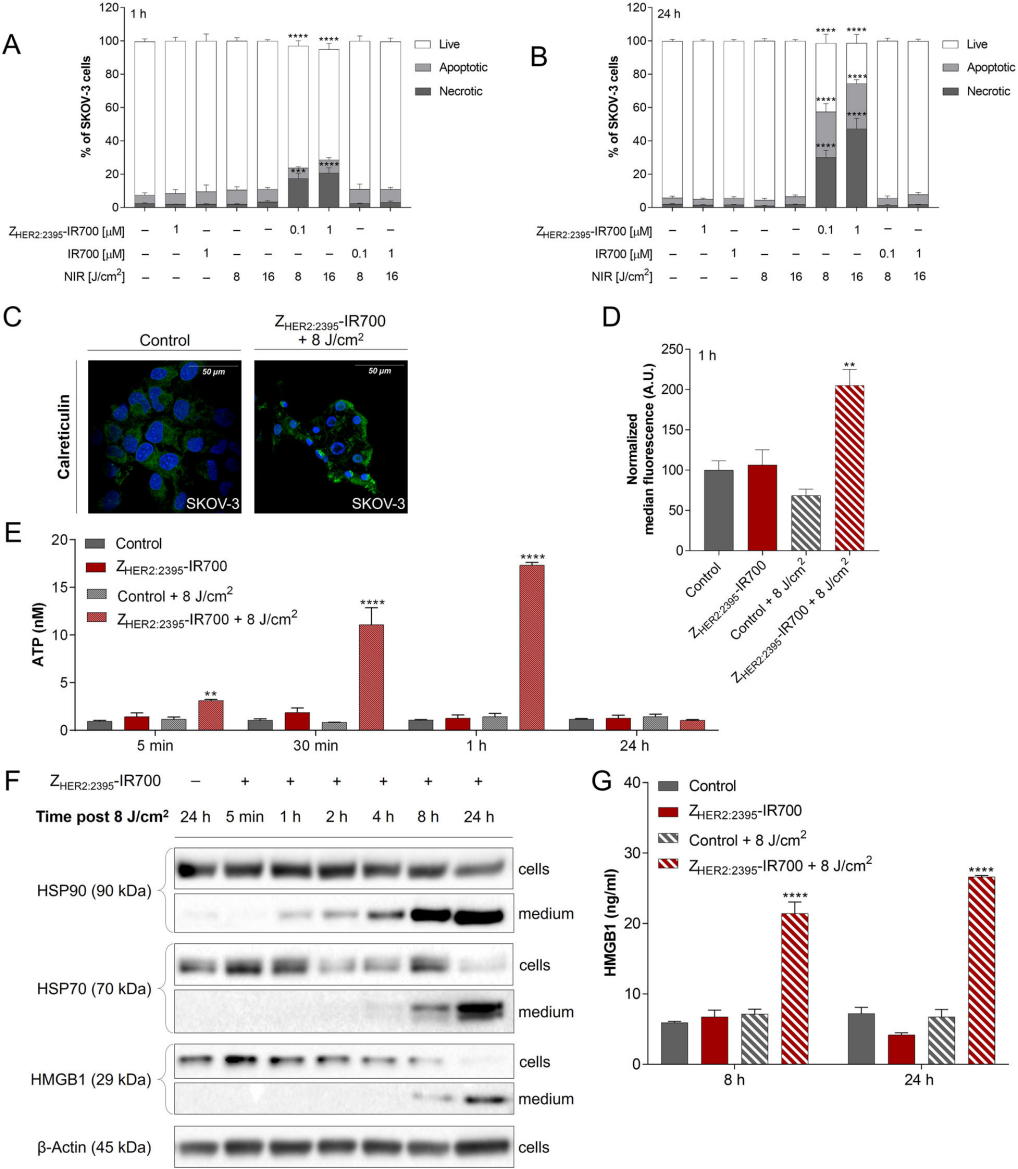
Immunogenic cell death (ICD) triggered by HER2 targeted phototherapy. **A**, **B** Changes in percentage of live, apoptotic and necrotic SKOV-3 cell populations measured 1 h (A) and 24 h (B) post-therapy, following 1 h incubation with the Z_HER2:2395_–IR700 (0.1 or 1 µM) or IR700 (0.1 or 1 µM) and exposure to 8 or 16 J/cm^2^ light dose. **C** Translocation of calreticulin (CRT) to the cellular membrane as illustrated by confocal microscopy, following immunofluorescence staining with anti-calreticulin-AlexaFluor405 antibody (overnight incubation in 4 °C) of methanol-fixed SKOV-3 cells untreated or 1 h post-phototherapy (0.1 µM Z_HER2:2395_–IR700 + 8 J/cm^2^). **D** Quantitative analysis of calreticulin translocation into the SKOV-3 cell membrane 1 h post-irradiation assessed by flow cytometry, following indirect immunofluorescence staining of live cells with primary rabbit anti-calreticulin antibody (30 min incubation in 4 °C) and secondary anti-rabbit-AlexaFluor®488 antibody (30 min incubation in 4 °C). **E** The amount of ATP released into the medium from SKOV-3 cells over time (5, 30 min, 1 or 24 h) post-treatment (1 h incubation with or without Z_HER2:2395_–IR700 (0.1 μM) with or without irradiation (8 J/cm^2^). **F** The expression level of HSP70, HSP90 and HMGB1 proteins in SKOV-3 cells and cell supernatants (medium) over time (30 min, 1, 2, 4, 8 or 24 h) post-treatment (0.1 μM Z_HER2:2395_–IR700 + 8 J/cm^2^) in comparison to irradiated control cells (8 J/cm^2^) assessed by Western blot. β-actin was used as loading control. **G** HMGB1 protein level released into the medium 8 h and 24 h post-treatment (0.1 μM Z_HER2:2395_–IR700 + 8 J/cm^2^) as measured by ELISA assay. All data are presented as mean ± SEM (*n* = 3). Statistical significance in comparison to the control (untreated) group was determined using ANOVA with Dunnett’s post hoc test. ****p ≤ 0.0001, ***p ≤ 0.001, **p ≤ 0.01.

**Fig. 4:**
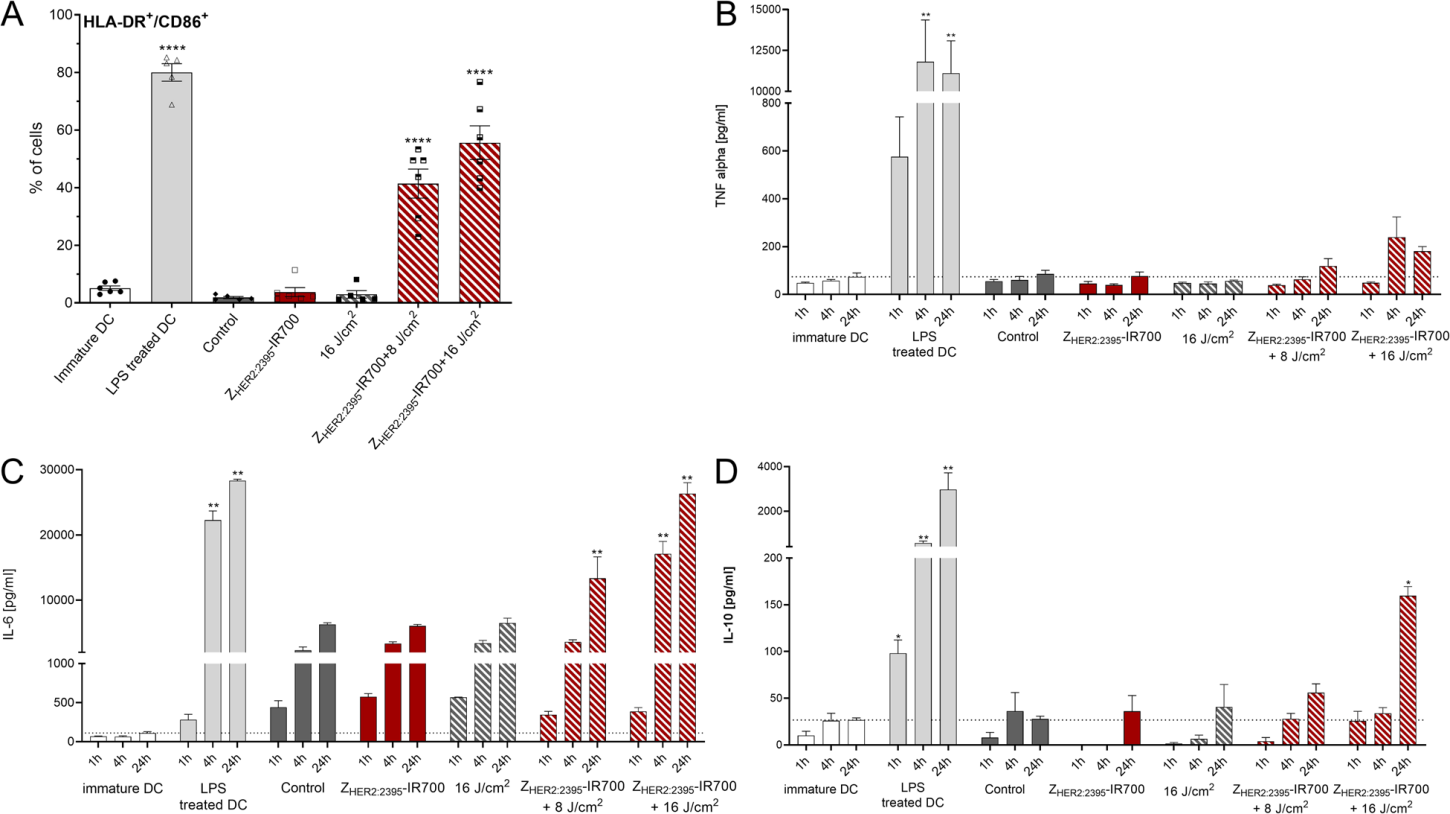
Co-culturing iDCs with ZHER2:2395–IR700-treated SKOV-3 cells post-irradiation leads to DCs maturation. Immature DCs (iDC; 5 days post-CD14+ monocytes isolation from PBMCs and differentiation with GM-CSF and IL-4) were cultured with SKOV-3 cells treated by HER2 affibody-based phototherapy (1 h incubation with 0.5 µM of Z_HER2:2395_–IR700 + irradiation with 8 or 16 J/cm^2^). iDCs cultured without stimulation were used as a negative control. E. coli lipopolysaccharide (LPS)-treated iDC were used as positive control for DC maturation. **A** Percenatge of double positive CD86^+^/HLA-DR + cells in DCs population 48 h post-treatment as assessed by flow cytometry. **B–D** TNF-α, IL-6 and IL-10 ELISA assays measuring cytokines levels in medium post-irradiation of iDCs co-cultured with SKOV-3 treated cells at different time points. Data are presented as mean ± SEM (*n* = 4–5). The graphs represent data from five healthy blood donors. Statistical significance in comparison to the iDC group was determined using ANOVA with Holm-Sidak correction test. **p ≤ 0.01, ***p ≤ 0.001, ****p ≤ 0.0001.

The treatment with either the conjugate, IR700 or light alone had little phototoxic effect on cell viability (Fig. 3A, B and Fig. S4A). Conversely, Z_HER2:2395_-IR700-based therapy induced cell death in both a light dose- and Z_HER2:2395_-IR700 concentration-dependent manner. The cell population shifted from viable to necrotic and the number of necrotic cells (Annexin V + /PI + ) peaked at 24 h post-irradiation (Fig. 3B and Fig. S4A), where ~47% of cells were dead when 1 μM of the conjugate and a light dose 16 J/cm^2^ were used, compared to ~2% of the untreated control. However, only ~30% of cells were necrotic when treated with 0.1 μM of the conjugate and a light dose 8 J/cm^2^. After 24 h, there was significant increase in the subpopulation of apoptotic cells (~28%), regardless of Z_HER2:2395_-IR700-based therapy regimen. Therefore, 0.1 μM of the conjugate and a light dose of 8 J/cm^2^ were selected as conditions for the subsequent experiments to investigate the generation of DAMPs in response to Z_HER2:2395_-IR700 phototherapy. Confocal microscopy images acquired 1 h post-treatment demonstrated redistribution of CRT within cellular membrane (Fig. 3C). Data were in agreement with the enhanced surface expression of CRT as measured by flow cytometry on live cells (Fig. 3D and Fig. S4B). Furthermore, there was a significant secretion of ATP as early as 5 min post-irradiation (Fig. 3E). A prominent release of HSP70/90 and HMGB1 from the cells to the medium following treatment with Z_HER2:2395_-IR700 was also detected by Western blot (Fig. 3F). Densitometric analysis confirmed that HSP70/90 and HMGB1 expression in medium markedly increases between 1 and 24 h (Fig. S4C). Consistent with these results, the expression level of HMGB1 in the medium, measured by an ELISA assay, reached the highest value (26.6 ± 0.4 ng/mL vs 7.2 ± 1.8 ng/mL for the control) at 24 h post-treatment initiation (Fig. 3G). No significant upregulation of DAMPs was detectable when light or conjugate-only treated-cells were studied. In addition, to verify the role of DAMPs in the maturation of DCs stimulated by Z_HER2:2395_-IR700 phototherapy, we co-cultured DCs with irradiated SKOV-3 cells for 48 h. Flow cytometry analysis showed that the expression of CD86 and HLA-DR molecules on the surface of DCs stimulated by LPS (positive control) or by Z_HER2:2395_-IR700 + 16 J/cm^2^ substantially and simultaneously increased compared to the negative controls (Fig. 4A and Fig. S5A–C). Furthermore, inflammatory cytokines including IL-6, TNF-α and IL-10 were elevated in supernatants from SKOV-3-DC co-cultures following treatment, as assessed by ELISA (Fig. 4B, D).

### Irradiation of tumours post-Z_HER2:2395_-IR700 treatment leads to strong inhibition of tumour growth

**Fig. 5:**
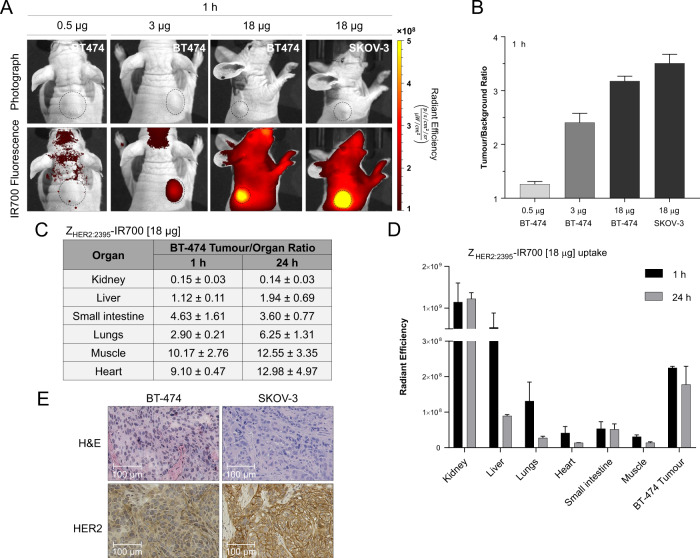
Z_HER2:2395_–IR700 binding specificity in vivo. **A** Fluorescence images of mice bearing HER2 + ve xenografts (BT-474 or SKOV-3) acquired 1 h after injecting different doses of Z_HER2:2395_-IR700 (0.5, 3 or 18 µg). **B** Tumour-to-background ratio calculated for varying Z_HER2:2395_–IR700 injected doses. **C**, **D** Z_HER2:2395_–IR700 (18 µg) uptake in excised tissues (1 and 24 h post-injection) and respective tumour-to-organ ratios. **e** HER2 and H&E staining of HER2-positive tumour sections indicating higher HER2 expression in SKOV-3 then BT-474 xenograft. All data are presented as mean ± SEM (*n* ≥ 3).

**Fig. 6:**
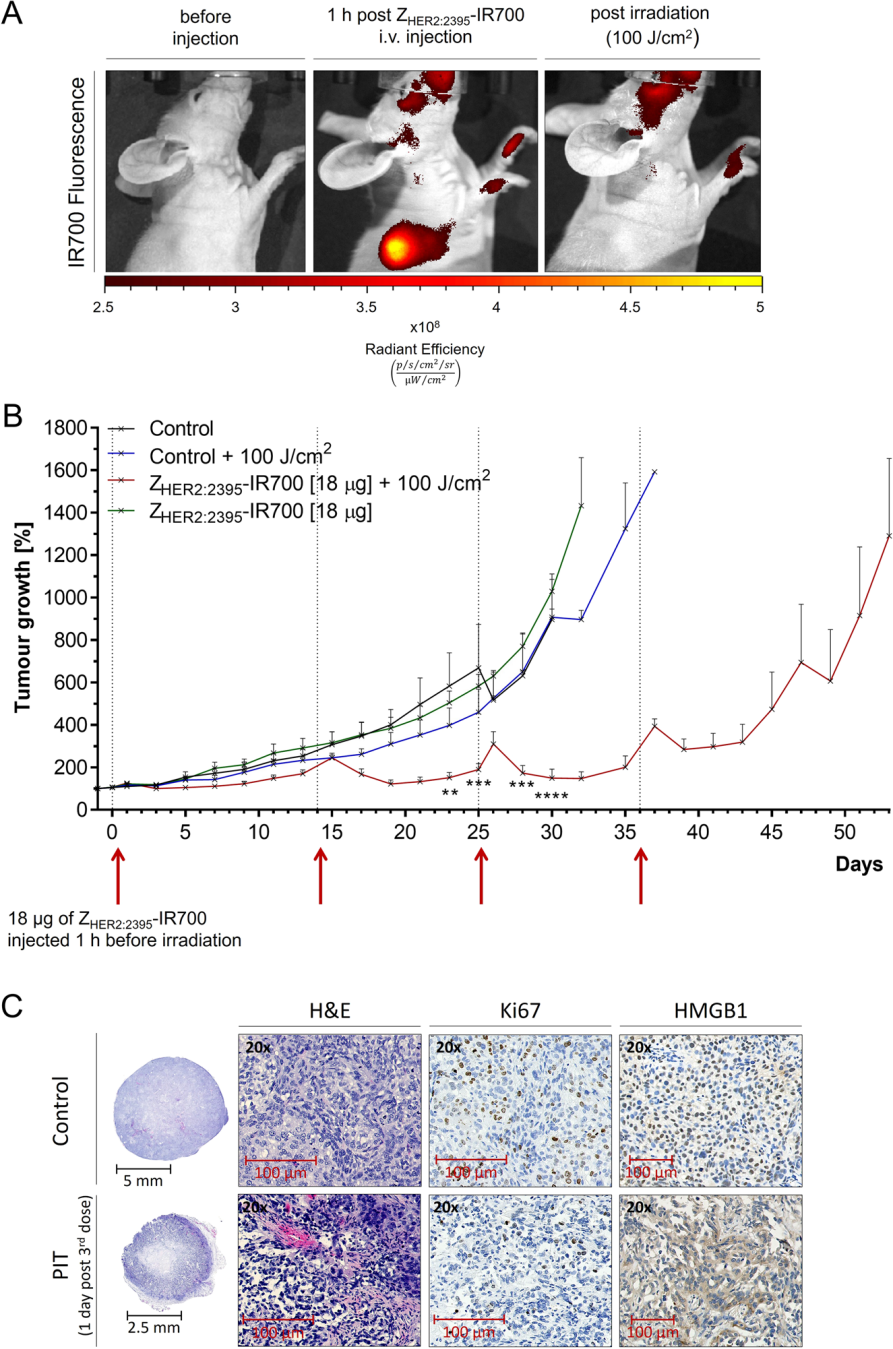
NIR light-based treatment using Z_HER2:2395_–IR700 proof-of-concept in vivo studies. **A** Fluorescence images of a mouse bearing a subcutaneous SKOV-3 tumour before, 1 h post-Z_HER2:2395_–IR700 (18 µg) intravenous injection and immediately after irradiation with a light dose of 100 J/cm^2^. **B** SKOV-3 tumour growth inhibition after four cycles of Z_HER2:2395_–IR700 phototherapy (18 µg Z_HER2:2395_–IR700 + 100 J/cm^2^) on day 0, 14, 25 and 36 in comparison to the control groups. Data are presented as mean ± SEM (*n* = 3 for each group). **p ≤ 0.01, ***p ≤ 0.001, ****p ≤ 0.0001 as assessed by unpaired multiple *t* tests with Holm-Sidak correction. **C** H&E, Ki67 and HMGB1 staining of SKOV-3 tumour sections collected after third cycle of the treatment. The images clearly show the presence of necrosis, decrease in cell proliferation and release of HMGB1 from the nuclei in the phototherapy-treated tumours.

To demonstrate the tumour-targeting efficacy of Z_HER2:2395_-IR700 in vivo, mice bearing subcutaneous BT474 tumours were injected intravenously with three different concentrations of the conjugate (0.5, 3 or 18 μg per mouse). The tumour fluorescence was monitored in vivo using an IVIS/Spectrum optical imaging system at different time points (Fig. S6A, B). 2D-fluorescence images acquired as early as 1 h post-Z_HER2:2395_-IR700 injection demonstrated clear delineation of tumours when 3 or 18 μg of the conjugate was administered (Fig. 5A and Fig. S6C). As shown in Fig. 5B, the injected conjugate quantity of 18 μg/mouse yielded a tumour-to-background ratio of 3.2. However, the semi-quantitative fluorescence signal intensities estimated from image ROIs slightly decreased over time to (1.00 ± 0.15) × 10^8^ (p/s/cm^2^/sr) × cm^2^/μW for a 3 μg/mouse dose and (3.35 ± 0.41) × 10^8^ (p/s/cm^2^/sr) × cm^2^/μW for a 18 μg/mouse dose at 24 h (Fig. S6A). Only negligible tumour uptake ((0.53 ± 0.11) × 10^8^ (p/s/cm^2^/sr) × cm^2^/μW; tumour-to-background ratio 1.24 ± 0.07) was found when the lowest dose 0.5 μg/mouse was injected (Fig. 5A, B and Fig. S6A–C). Of note, no fluorescence signal was observed in mice injected with saline (Fig. S6D). Importantly, the imaging data were corroborated by ex vivo biodistribution studies that showed a tumour uptake of (2.25 ± 0.04) × 10^8^ (p/s/cm^2^/sr) × cm^2^/μW (tumour-to-muscle ratio of 10.17 ± 2.76) 1 h post-injection and (1.78 ± 0.51) × 10^8^ (p/s/cm^2^/sr) × cm^2^/μW at 24 h (tumour-to-muscle ratio 12.55 ± 3.35) (Fig. 5B and Fig. S6C). Furthermore, as shown in Fig. 5C, intense fluorescence signal was detected in the kidney at 1 and 24 h post-injection, which is consistent with the known excretion profile of affibody molecules^26^. In addition, uptake in the liver and lungs gave an initial strong fluorescence signal, which markedly decreased at 24 h (Fig. 5C, D and Fig. S6E). This may be attributed to the lipophilicity of the dye. The heart and small intestines showed fluorescence signal just above the muscle level (background). In parallel, we also injected a group of SKOV-3 xenografts (*n* = 6) with 18 μg/mouse of the conjugate. Since, the measured radiant efficiency attributed to Z_HER2:2395_-IR700 prominently increased 1 h post-injection ((6.20 ± 0.33) × 10^8^ (p/s/cm^2^/sr) × cm^2^/μW), the SKOV-3 tumour model and 1 h time point were selected for subsequent Z_HER2:2395_-IR700-mediated phototherapy studies (Fig. 5A, B and Fig. S6C). Importantly, the tumour uptake in both xenograft models correlated with the HER2 expression level as assessed ex vivo by IHC staining (Fig. 5E). After confirming conjugate uptake in vivo, we hypothesised that phototherapy with Z_HER2:2395_-IR700 would lead to significant delay in tumour growth. Following SKOV-3 tumour establishment (volume of ~50 mm^3^), mice were randomly divided into four groups (day 0) (i) control (no treatment), (ii) 100 J/cm^2^, (iii) Z_HER2:2395_-IR700 (18 μg) and (iv) Z_HER2:2395_-IR700 (18 μg) + 100 J/cm^2^. Irradiating tumours with 100 J/cm^2^ led to complete photobleaching of the fluorescence signal indicating that a sufficiently high light dose had been delivered (Fig. 6A). After the first treatment dose, tumour growth rate and body weight were measured every second day (Fig. 6B and Fig. S7A). Due to the slight increase in tumour volumes in the group treated with Z_HER2:2395_-IR700 and light on days 14, 25 and 36, all the groups received a top-up treatment dose (Fig. 6B). Compared with either the control or Z_HER2:2395_-IR700 group alone, Z_HER2:2395_-IR700-based phototherapy significantly inhibited tumour growth (p < 0.05, control group vs. Z_HER2:2395_-IR700/100 J/cm^2^) over a period of 32 days (Fig. 6B). Neither skin necrosis nor systemic toxicity was observed within any group. Of note, mice from the control groups had to be sacrificed earlier because their tumours exceeded size limits specified by institutional guidelines and the animal project licence. Importantly, mice treated with Z_HER2:2395_-IR700-based phototherapy showed significantly higher median survival as compared to controls (Fig. S7B). Ex vivo analysis of tumour sections demonstrated distinct differences in tissue structure between control and treated mice, confirming that irradiation of Z_HER2:2395_-IR700 led to extensive tumour necrosis (Fig. 6C). Ki-67 staining revealed a reduced cell proliferation index, especially on the margins of the treated tumours. Furthermore, in the control tumours we found only strong nuclear HMGB1 staining, whereas in the treated tumours HMGB1 was localised in the nuclei and cytoplasm (Fig. 6C).

## Discussion

The development of tumour-targeted treatments that can drive immunogenic cancer cell death and influence the innate and adaptive anti-tumour immune response are of paramount importance. Several recent studies have demonstrated that PDT and mAb-based PIT can convert a ‘cold’, relatively immunosuppressive tumour microenvironment into a ‘hot’, inflammatory, immunogenic one, thereby increasing susceptibility to ICPIs^27,28^. Therefore, we investigated whether phototherapy using IR700-based HER2-targeted affibody conjugate could (i) promote the release of DAMPs capable of inducing maturation of DCs in vitro and, (ii) delay tumour growth in receptor-positive xenografts. For that purpose, the phthalocyanine dye IR700 was conjugated to affibody molecule Z_HER2:2395_. The high binding affinity (nM range) to HER2, the small size and consequent great tumour penetration of Z_HER2:2395_ make it an ideal targeting vector. Moreover, as Z_HER2:2395_ binds to a different HER2 epitope than therapeutic anti-HER2 mAbs, it would allow for their simultaneous application in future combinatorial treatment strategies. We initially confirmed the specificity of Z_HER2:2395_-IR700 binding to HER2-expressing cancer cells. Additionally, as high levels of ROS can elicit oxidative stress and cellular damage^29^, we demonstrated the conjugate’s ability to produce ROS and singlet oxygen upon light irradiation. The treatment data showed a conjugate concentration- and light dose-dependent decrease in cell viability in response to Z_HER2:2395_-IR700 phototherapy, whereas only negligible cell death was observed when dye, Z_HER2:2395_-IR700, or light alone were applied. This clearly indicated that the target-specific binding of the conjugate combined with light treatment are required to induce cancer cell death. Consistent with other reports, we observed rapid necrotic cell death upon conjugate irradiation, as evidenced by bleb formation, cellular swelling and membrane rupture^30,31^. Interestingly, Sato et al. have recently reported that photochemical reactions following irradiation of mAb-based IR700 conjugates may lead to a release of hydrophilic side chains of IR700 which changes the complex structure making the remaining molecule insoluble. This subsequently reduces cell membrane integrity due to the damage to transmembrane target proteins and allows the surrounding aqueous fluid to flow into the cell^17^. Whether, the exact same photochemical reaction might induce cell death post-NIR light irradiation of Z_HER2:2395_-IR700 will need to be investigated. However, the inhibition of cell death we observed when pre-treating the cells with ROS or apoptosis inhibitors seem to indicate multiple co-existent cell death mechanisms in response to Z_HER2:2395_-IR700 phototherapy.

Importantly, Z_HER2:2395_-IR700 followed by exposure to a single dose of NIR light in vitro triggered the rapid activation of stress markers including translocation of CRT to the cell surface in SKOV-3 cells, as well as the secretion of ATP, HSP70/90 and HMGB1 into the medium. This was in line with previously published studies demonstrating that PDT and PIT induce ICD^7,8,32^. Given the fact that DAMPs exposed at the surface of dying cells result in DC activation and maturation as a prelude to priming anti-tumour adaptive T cell responses^33^, we differentiated peripheral blood mononuclear cells (PBMC) into immature DCs and subsequently co-cultured these cells with Z_HER2:2395_-IR700 pre-treated cancer cells. Augmented expression levels of DC markers, including CD86 and MHC II (e.g. HLA-DR), were observed when DCs were co-cultured with SKOV-3 cells post-Z_HER2:2395_-IR700 phototherapy, indicating enhanced DC maturation. An increase in the level of inflammatory cytokines confirmed functional stimulation of DC. Importantly, no changes were detected in these markers when DCs were co-cultured with untreated or irradiated SKOV-3 cells in the absence of Z_HER2:2395_-IR700 conjugate. Taken together, these in vitro results suggest that Z_HER2:2395_-IR700 phototherapy rapidly leads to ICD via cell death mechanisms (necrotic and/or apoptotic) that are associated with effective activation of DCs. In the case of necrosis, the cell membrane becomes damaged, resulting in the release of cellular contents into the extracellular space, and subsequent initiation of the inflammatory response that attracts antigen-presenting cells^34^. During apoptosis, on the other hand, these products are packaged into membrane-bound structures that can be consumed by DCs. Those, in turn, are capable of cross-presenting antigens to the immune system for the induction of T cell immunity^35,36^. Therefore, the relationship between the mode of cancer cell death and the efficient induction of an anti-tumour immune response by phototherapy-based regimens is important.

Having successfully confirmed therapeutic effect of the conjugate in vitro, the response to Z_HER2:2395_-IR700 in vivo was investigated. A distinct fluorescent signal was observed in tumours as early as 1 h after i.v. injection of Z_HER2:2395_-IR700, indicating the conjugate’s strong targeting ability. Subsequently, we measured tumour uptake post-administration of three escalating doses of the conjugate, as studies reported by another group showed that increasing doses of mAb-IR700 correlated with greater treatment efficacy^12^. However, the use of high amounts of targeted agent may sometimes saturate receptors of interest, leading to non-specific binding in tissues surrounding the tumour. In this study, the relatively small molecular size of the affibody molecule and the rapid clearance of unbound conjugate ensured that high tumour-to-background contrast images were seen even at the highest investigated dose (18 μg) just 1 h post-injection. In addition, biodistribution studies showed that the conjugate is still retained in the tumour at 24 h post-injection, which highlights the potential for its therapeutic applications. Apart from the tumour, increased uptake of the conjugate was found in the kidneys, which is associated with renal elimination and re-absorbance of the affibody^26^. Afterwards, we demonstrated that Z_HER2:2395_-IR700-mediated phototherapy, but not light or Z_HER2:2395_-IR700 alone, led to a significant inhibition of SKOV-3 tumour growth and prolonged overall survival of mice from 30 to 52 days compared to vehicle control group. When Mitsunaga et al. performed a similar experiment using a one-off mAb-IR700 PIT treatment in vivo, significant target-specific tumour cell death was initially observed followed by tumour recurrence^20^. In order to enhance the effectiveness of Z_HER2:2395_-IR700, we based our treatment schedule on regular monitoring of tumour volume and delivering further phototherapy doses when the increase in volume was measured to be ∼15%. We observed more necrotic regions in the group exposed to Z_HER2:2395_-IR700 and light than in the control group, as indicated by H&E staining. The additional dosing resulted also in a subsequent, yet short-term arrest in tumour growth. Interestingly, we found that after each therapy cycle the tumour volume temporarily increased. We speculate that the rapid cell death could lead to cytotoxic oedema, in which extracellular water passes into the cells resulting in their swelling.

In conclusion, we demonstrated that Z_HER2:2395_-IR700 followed by exposure to NIR light led to HER2-specific cell death, consequent release of DAMPs and in vitro activation of DCs. Moreover, following treatment of HER2-positive xenografts, delayed tumour growth and increased median survival was observed.

Further studies using syngeneic mouse models will be needed to investigate the capability of affibody-IR700 mediated phototherapy to activate and expand tumour-reactive cytotoxic T lymphocytes, which could provide clear indications of immunomodulatory activity of the conjugate and rationale for combinatorial approaches using affibody-based PIT with ICPIs. Furthermore, owing to the rapid and specific accumulation of Z_HER2:2395_-IR700 in HER2-positive tumours, the conjugate could be used in the clinic as a tool for both NIR fluorescence image-guided tumour resection and simultaneous light irradiation-mediated elimination of residual cancer cells as a trigger to activate the host anti-tumour immune responses. Such an approach could be an attractive alternative, particularly for patients whose tumours acquire resistance to conventional anti-HER2 therapies.

## Methods

### Preparation of Z_HER2:2395_-IR700

IRDye700DX–maleimide (IR700; ex. 689 nm, em. 700 nm; LI-COR Biosciences, US) was conjugated to the affibody molecule Z_HER2:2395_-Cys (supplied by AffibodyAB, Sweden) as previously described^14^. Some technical details are additionally given in the Supporting Information.

### Cell lines

Breast (BT-474, MDA-MB-175, MDA-MB-231, and MDA-MB-468) and ovarian (SKOV-3) cancer cell lines were obtained from the American Type Tissue Culture Collection (ATCC, US). All cell lines were tested and authenticated by short tandem repeat (STR) DNA profiling analysis and confirmed to be mycoplasma-free. BT-474, MDA-MB-468 cells were cultured in RPMI-1640 (Gibco, Life Technologies, US) and MDA-MB-175, MDA-MB-231, SKOV-3 cells in DMEM (Gibco, Life Technologies, US), supplemented with 10% heat-inactivated fetal bovine serum (FBS, Gibco, Life Technologies, US) and maintained at 37 °C in a humidified atmosphere supplied with 5% CO_2_. The 3D-spheroids were grown in ultra-low attachment (ULA; Corning, Germany) 96-well round bottom plates as previously described^37^.

### Binding specificity in vitro

To assess the expression level of HER2 and validate the binding specificity of Z_HER2:2395_-IR700 in vitro, flow cytometry was performed. A detailed description of the procedure is provided in the Supporting Information.

### Cellular accumulation of Z_HER2:2395_-IR700

To test the targeting specificity and internalisation of the conjugate, either MDA-MB-468 or SKOV-3 cells (2 × 10^5^) were plated on confocal glass-bottomed dishes (MatTek, US) and incubated in a complete medium with Z_HER2:2395_-IR700 (1 µM) for 1 h at 4 °C or 1, 4 and 6 h at 37 °C, respectively. To investigate the penetration of the conjugate, SKOV-3 3D spheroids were incubated either with Z_HER2:2395_-IR700 (1 µM) or anti-HER2-FITC mAb (1 µM, sc-23864, Santa Cruz Biotechnology, US) for 6 h in 37 °C. The detailed imaging and data analysis protocols are given in the Supporting Information.

### In vitro treatment studies

The photocytotoxicity of either Z_HER2:2395_-IR700 (0.01–1 µM) or free IR700 (1 µM) was measured using CellTiter-Glo^®^ luminescent assay (Promega, UK), on 2D and 3D cell cultures following 6 h incubation and irradiation (16 J/cm^2^). Further experimental details are given in the Supporting Information.

### Annexin V/PI assay

At 1 and 24 h post-irradiation, cells were resuspended in 0.1 mL of propidium iodide (PI; 0.5 μg/mL) and Annexin V-AlexaFluor™488 (AnnexinV/Dead Cell Apoptosis Kit, Thermo Fisher Scientific, US) and subsequently analysed using BD™ LSRII flow cytometer. A detailed description of the procedure is given in the Supporting Information.

### Singlet oxygen and ROS detection

To evaluate singlet oxygen (^1^O_2_) and ROS production in response to phototherapy, SKOV-3 cells were incubated with either the cell permeant reagent 2’,7’-dichlorofluorescein diacetate (DCFDA; Cellular Reactive Oxygen Species Detection Assay Kit; Abcam, UK) or SOSGR (Singlet Oxygen Sensor Green Reagent; Molecular Probes, US) reagents with or without Z_HER2:2395_-IR700 (1 μM), IR700 (1 μM) or medium alone for 6 h at 37 °C. Post-light irradiation the fluorescence was measured according to the manufacturer’s protocol using a FLUOstar Omega microplate reader (ex. filter: 485 nm, em. filter: 520 nm; BMG Labtech, Germany).

### Western blot

Cell lysates were prepared as previously described^38^. A complete list of used antibodies and the detailed methods are provided in the Supporting Information.

### Measurement of ATP secretion

In order to determine ATP secretion post-phototherapy, the ENLITEN^®^ test (Promega, US) was used. SKOV-3 cells were seeded in cell-culture dishes and treated as described in the above section “*Annexin V/PI assay”*. The medium from each dish was collected at 5 min, 30 min, 1 h and 24 h post-light irradiation and centrifuged at 500×*g* for 5 min at 4 °C. The supernatants were used to determine the extracellular ATP concentration according to the manufacturer’s instruction.

### Measurement of HMGB1 release

To quantify HMGB1 released from SKOV-3 cells post- Z_HER2:2395_-IR700 irradiation, culture supernatants from indicated cells were collected and centrifuged at low speed (500×g for 5 min at 4 °C) to clear cell debris. HMGB1 concentration was measured using an ELISA kit (Tecan, IBL International, Germany) per manufacturer’s guidelines.

### Monitoring calreticulin translocation

To visualise calreticulin membrane translocation via confocal microscopy, methanol-fixed control or treated (0.1 µM Z_HER2:2395_-IR700 + 8 J/cm^2^) SKOV-3 cells were blocked with 5% bovine serum albumin and incubated overnight with anti-calreticulin mAb conjugated to Alexa Fluor®405 (ab210431, Abcam, UK). To measure calreticulin membrane exposition post-phototherapy (0.1 µM Z_HER2:2395_-IR700 + 8 J/cm^2^) cells were collected and incubated with anti-calreticulin mAb (1:100; PA3-900, Thermo Fisher Scientific, US) for 30 min at 4 °C, followed by 30 min incubation with secondary Alexa-Fluor488-labeled mAb (1:1000, A-11008, Thermo Fisher Scientific, US). Only viable cells (PI negative) were taken for flow cytometry analysis.

### Dendritic cells co-culture

Perpheral blood mononuclear cells (PBMCs) were isolated from leukocyte cones (NC24) obtained from healthy donors supplied by the NHS blood and transplant service (UK) by Ficoll (GE Healthcare, US) gradient centrifugation (according to MACS Miltenyi Biotec protocol). CD14+ monocytes were seperated from PBMCs via magnetic positive selection using CD14+ beads and MACS column (Miltenyi Biotec, Germany). Immature dendritic cells (iDC) were culture in RPMI medium supplemented with FBS, interleukin-4 (IL-4, 500 U/ml; PeproTech, US) and granulocyte-macrophage colony-stimulating factor (GM-CSF, 800 U/ml; PeproTech) for 5 days. Next, the co-culture of iDC and Z_HER2:2395_-IR700-treated SKOV-3 cells (ratio 1:2 iDC/cancer cell) was irradiated (16 J/cm^2^). DC maturation was induced by stimulating the iDC with E.coli lipopolysaccharide (LPS, 100 ng/ml; Sigma) for 12 h (a positive control). After 48 h, all floating cells were collected and with Pacific Blue™ anti-human CD14 (#367121), FITC anti-human CD86 (#374204) and APC anti-human HLA-DR (#307610) (all antibodies from BioLegend, US) and PI. DC maturation was evaluated on single, live (PI-) and CD14- gated cells populations by flow cytometry. Cell supernatants were collected at different time points and used for anti-IL6, anti-IL-10 and anti-TNF-α ELISA assays according to manufacture protocols (BioLegend, US).

### Animals

All animal experiments were conducted in compliance with licences issued under the UK Animals (Scientific Procedures) Act 1986 and following local ethical review. Studies were compliant with the United Kingdom National Cancer Research Institute Guidelines for Animal Welfare in Cancer Research^39^. Female NSG nude mice (6–8 weeks old) obtained from the in-house breeding colony were used in the animal studies.

### In vivo biodistribution and therapy

To evaluate Z_HER2:2395_-IR700 accumulation and efficacy post-irradiation in vivo, mice bearing subcutaneous SKOV-3 or BT-474 tumours implanted on the right shoulder (7 × 10^6^ cells/0.1 mL PBS/Matrigel; 20% v/v%; BD Matrigel™ Matrix, BD Bioscience, US) were used. When the tumours reached ~50–100 mm^3^, mice were injected i.v. with Z_HER2:2395_-IR700 (0.5, 3 or 18 µg) and imaged at the indicated time points using the IVIS/Spectrum imaging system (ex. filter: 675 nm, em. filter: 720 nm; PerkinElmer, US). Regions of interest (ROIs) were drawn around the tumours and background tissue, and the average radiant efficiency ((p/s/cm^2^/sr) × cm^2^/μW) in each ROI was calculated. To determine the conjugate distribution, mice were sacrificed by cervical dislocation at 1 and 24 h post-injection of Z_HER2:2395_-IR700 and major tissues and tumours were collected for ex vivo fluorescence imaging. The tumour-to-organ ratios were determined and used to select the optimal conditions for the following treatment studies. For proof of concept therapy studies, mice (*n* = 5 per group) were randomly assigned to the following groups: (i) no treatment, (ii) light exposure only (100 J/cm^2^), (iii) Z_HER2:2395_-IR700 (18 µg) without light exposure, (iv) Z_HER2:2395_-IR700 (18 µg) with light exposure (100 J/cm^2^). The detailed treatment and biodistribution protocol is given in the Supporting Information.

### Immunohistochemical analysis

Spheroids submerged in agar and paraformaldehyde-fixed tumours were embedded in paraffin, sectioned into 4–5 μm-thick slices, and mounted on microscope slides. The detailed staining procedures with the various antibodies are described in the Supporting Information.

### Statistical analysis

Unless otherwise stated, data were expressed as the mean ± SEM. Statistical significance, sample size calculations and correlation analysis are described in detail in the Supporting Information.

## Supplementary information

Supplementary Materials and Menthods

Supplementary Figure Legends

Supplementary Figure 1

Supplementary Figure 2

Supplementary Figure 3

Supplementary Figure 4

Supplementary Figure 5

Supplementary Figure 6

Supplementary Figure 7
