# Supplementary material for: Immunomodulatory activity of IR700-labelled affibody targeting HER2
Source: Cell Death Dis. 2020 Oct 20;11(10):886. doi: 10.1038/s41419-020-03077-6 (PMC7576828; doi:10.1038/s41419-020-03077-6)
Supplement: Supplementary file 1 — Supplementary Materials and Menthods [file 41419_2020_3077_MOESM1_ESM.docx]

**SUPPLEMENTARY MATERIALS AND METHODS**

**Preparation of Z_HER2:2395_-IR700**

Briefly, Z_HER2:2395_-Cys was incubated with Tris(2-carboxyethyl)phosphine hydrochloride (TCEP^.^HCl, Sigma-Aldrich, UK) (25-fold molar excess) and IR700 (8-fold molar excess) in 1M Phosphate buffer pH 7.2 for 2.5 h at 40°C. The product was isolated by size-exclusion chromatography using two consecutive Zeba™ Spin Desalting Columns (7 kDa MWCO) (Thermo Fisher Scientific, UK). The conjugate was characterized by ESI-MS using a 6520 Series qToF mass spectrometer fitted with a dual ESI ionization source (Agilent, Santa Clara, USA) and Tricine-SDS-PAGE. On the gel, the protein bands were visualized after silver staining (Silver Staining kit, Thermo Scientific, UK) using a ChemiDoc™XRS System (Bio-Rad, UK). Fluorescent bands were captured using a Typhoon™ FLA7000 scanner (ex. 635 nm, band filter 670 nm; GE Healthcare Life Sciences, UK).

**Binding specificity *in vitro***

To assess the expression level of HER2 and validate the binding specificity of Z_HER2:2395_-IR700 *in vitro*, flow cytometry was performed. Cells (3×10^5^) were incubated for 1 h at 4°C with FITC-labelled mAb against HER2 (1 mg/10^6^ cells; sc-23864, Santa Cruz Biotechnology, US), IR700 (30 nM), Z_HER2:2395_-IR700 (30 nM) with or without 50-fold excess of unlabelled Z_HER2:2395_. Post-incubation, cells were washed twice with cold phosphate-buffered saline (PBS) and resuspended in 400 µL of PBS for data acquisition. For each measurement, data from 10 000 events were collected with a BD™ LSRII flow cytometer (BD Biosciences, US) using 488 nm and 635 nm lasers. Fluorescence of FITC and IR700 was detected by 530/30 nm and 730/45 nm (Alexa700 setting) bandpass filters, respectively. Signals from single cell-populations were gated and analysed using FlowJo software (FlowJo LLC, US). Each group was normalized to the median fluorescence intensity (MIF) acquired for SKOV-3 cells.

**Cellular accumulation of Z_HER2:2395_-IR700**

Hoechst®33342 (nuclear stain; 5 µg/mL; Thermo Fisher Scientific, US) and Lysotracker™ Green DND-26 (lysosome stain; 5 nM; Thermo Fisher Scientific, US) were used for cellular counterstaining 30 min before imaging. Samples were washed twice post-incubation with PBS and images were captured using the Zeiss LSM700 confocal microscope (Carl Zeiss Inc, Germany) equipped with 405 nm, 488 nm and 639 nm lasers and appropriate filters. Data were analysed using the Zen2009 software (Carl Zeiss Inc, Germany).

***In vitro* PIT studies**

SKOV-3, BT-474 or MDA-MB-468 cells were seeded (8×103) in black, cell-culture 96-well plates with clear bottoms 24 h before the experiment. For 3D SKOV-3 spheroids, ~3×103 cells were seeded in ULA plates and left to grow for 96 h. Afterwards, fresh medium (control cells) and medium containing either ZHER2:2395-IR700 (0.01-1 µM) or IR700 (1 µM) was added for 6 h and the plates were incubated at 37°C. Next, cells or spheroids were rinsed twice with phenol-red-free medium and irradiated (16 J/cm2) using a LED light source (L690−66−60, Marubeni America Co., US) at wavelengths of 670–710 nm (peak at 690 nm, mean power density of 12.5 mW/cm2, current of 285 mA). For inhibition studies SKOV-3 cells were co-incubated (6 h pre and 24 h post irradiation) with NAC (5 mM) or Z-VAD-FMK (25 µM). The viability of cells and 3D spheroids was assessed by the CellTiter-Glo® luminescent assay at 24 or 96 h post-irradiation, respectively. Data are presented as the mean of n = 3 independent measurements ± SEM. Spheroid size was measured using the Celigo® imaging system (Nexcelom Bioscience, US). In addition, the live/dead cells within the 3D spheres were visualized using a LIVE/DEAD™ Cell Imaging Kit (Thermo Fisher Scientific, UK) 96 h post-treatment according to the manufacturer's protocol. Briefly, SKOV-3 spheroids were co-incubated with Calcein AM (live, green; 488/515 nm) and ethidium homodimer-1 (EthD-1, dead, red; 570/602 nm) for 1 h. Afterwards, the spheres were washed with PBS and images were captured with the Zeiss LSM700 confocal microscope (Carl Zeiss Inc). Maximum intensity projections (MIP) were generated from Z-stack images taken at 6-7 μm intervals, using the following settings: 1024x1024 pixels, 8 speed, 4 averaging.

**Annexin V/PI assay**

SKOV-3 cells (3×10^5^) were seeded in 35 mm cell-culture dishes and cultured overnight. On the following day, the medium was replaced with either Z_HER2:2395_-IR700 (0.1 or 1 µM), IR700 (0.1 or 1 µM) or medium alone and cells were incubated further at room temperature (RT) for 1 h or 37°C for 6 h. PIT experiments were performed using a mean optical fluence of either 8 or 16 J/cm^2^. At 1 and 24 h post-irradiation, cells were dissociated with trypsin (Gibco, Life Technologies, US) and resuspended in 0.1 mL of Annexin V-binding buffer with propidium iodide (PI; 0.5 mg/mL) and Annexin V-AlexaFluor™488 (AnnexinV/Dead Cell Apoptosis Kit, Thermo Fisher Scientific, US) according to the manufacturer's instruction. The sample was then incubated for 15 minutes at RT, protected from light. Stained cells were analysed using BD™ LSRII flow cytometer, using green fluorescence emission at 530/30 nm bandpass (for Annexin V-AlexaFluor™488) and 610/20 nm bandpass (for PI). For analysis, the different cell populations were discriminated on the basis of the labelling: viable (Annexin V–/PI–), apoptotic (Annexin V+/PI–) and necrotic (Annexin V+/PI+).

**Western blot**

Proteins released into the medium were extracted using an acetone precipitation protocol (Thermo Fisher Scientific, US). Samples were resolved by Tris-SDS/PAGE and transferred onto a nitrocellulose membrane (GE Healthcare, UK). The blots were blocked with 5% milk in Tris-buffered saline with Tween (0.05%) for 1 h at RT and then incubated overnight at 4°C with primary antibodies, followed by 1 h incubation at RT with horseradish peroxidase-conjugated secondary antibody. The primary antibodies used were against HER2 (1:1000, #2242), GAPDH (1:1000, #5174), HSP70 (1:800#4872), HSP90 (1:800, #4877), HMGB1 (1:500, #6893) and β-actin (1:1000, #4967), all from Cell Signalling Technology, US. Densitometric analysis results are presented as the mean + SEM (*n* = 3 independent experiments).

***In vivo* biodistribution and therapy**

All procedures were carried out under an approved Home Office project license (number PPL PCC916B22). Female NSG nude mice (6-8 weeks old) obtained from the in-house breeding colony were used. Animals were housed in 500 square cm Allentown Nexgen cages and given access to mice maintenance food (Labdiet, US) and water ad libitum. Environmental conditions were a temperature of 21^o^C±1^o^C, humidity of 55%±10%, and a 12:12 light:dark cycle with lights. Environmental enrichment included bedding substrate of Corn Cob 6/8, Aspen chew stick 5x1x1 cm (Datesand, product code CS3C15) and one Bed r’nest paper wool nesting material (Datesand, Product code BEDRNEST). During housing, animals were monitored twice daily for health status. No adverse events were observed.

To evaluate Z_HER2:2395_-IR700 accumulation and PIT efficacy *in vivo*, mice bearing subcutaneous SKOV-3 or BT-474 tumours implanted on the top right shoulder (7×10^6^ cells/0.1 mL PBS/Matrigel; 20% v/v%; BD Matrigel™ Matrix, BD Bioscience, US) were used. When the tumours reached approximately 50-100 mm^3,^ mice were injected intravenously via the tail vein with 100 mL of Z_HER2:2395_-IR700 (0.5, 3 or 18 µg) in 0.9% sterile saline, anaesthetised using isoflurane (1.5%-2% v/v in O_2_, and imaged at the indicated time points using the IVIS/Spectrum imaging system (ex. filter: 675 nm, em. filter: 720 nm; PerkinElmer, US). Regions of interest (ROIs) were drawn around the tumours and background tissue, and the average radiant efficiency ((p/sec/cm^2^/sr)×cm^2^/mW) in each ROI was calculated. Mice were sacrificed by cervical dislocation at 1 and 24 h post-injection of Z_HER2:2395_-IR700 and major tissues and tumours were collected for *ex vivo* fluorescence imaging to determine the conjugate distribution. The tumour-to-organ ratios were determined and used to select the optimal conditions for the following PIT studies. For proof of concept therapy studies, mice (n = 4-5 per group) were randomly assigned to the following groups: i) no treatment, ii) light exposure only (100 J/cm^2^), iii) Z_HER2:2395_-IR700 (18 µg in 100 mL 0.9% sterile saline) without light exposure, iv) Z_HER2:2395_-IR700 (18 µg in 100 mL 0.9% sterile saline) with light exposure (100 J/cm^2^).

The last group was subjected to 4 cycles of therapy on days 0, 14, 25 and 36. The tumours were exposed to LED light for a duration of 516 seconds, which corresponded to a total dose of 100 J/cm^2^ (mean power density of 0.19 ± 0.01 W/cm^2^, LED current = 300 mA). During irradiation, the surrounding normal tissues were protected with aluminium foil. Following first and third dose one mouse from each group was sacrificed by cervical dislocation, tumours excised and evaluated *ex vivo.* Mice were weighted and tumour volumes measured every day by calliper using the formula (L × H × W)/2, where L is the length, W is the width, and H is the height of the tumour in millimetres. Mice were euthanized by cervical dislocation when the tumours exceeded volume of 500 mm^3^.

**Immunohistochemical analysis**

Spheroids submerged in agar and excised tumour tissues were prepared for immunohistochemistry (IHC) as follows: briefly, samples were collected and fixed in freshly prepared 4% paraformaldehyde. Following paraffin-embedding and sectioning (4-5 µm slices), they were stained with hematoxylin and eosin (H&E, Sigma-Aldrich), anti-Ki67 (1:200, #9027, Cell Signalling, US), anti-HMGB1 (1:1000, ab18256, Abcam, US) and anti-HER2 (1:200, #2242, Cell Signalling, US) with the use of VECTASTAIN^®^ ABC HRP Kit (Vector Laboratories, US).

**Statistical analysis**

If not otherwise stated, three experimental replicates were performed for the *in vitro* studies. All data are presented as mean ± SEM. For *in vivo* assessment of Z_HER2:2395_-IR700 distribution and PIT treatment response n = 3-5 mice per group were used. The mice were randomized before treatment initiation. No studies were blinded. No data were excluded from the analysis. Group differences were assessed by unpaired multiple t tests with Holm-Sidak post-hoc tests for measuring significance. Results were considered statistically significant when p<0.05. For significant differences, * means p<0.05, ** p<0.01 and *** p<0.001, **** p<0.0001. Survival analysis was conducted with Kaplan-Meier curves, and their comparison was performed with the log-rank (Mantel-Cox) test. The number of experiments performed (minimum 3) is detailed in each Figure (n). Statistical analysis was performed using Prism software (GraphPad Prism Software v8.0).
