# Supplementary material for: Immunomodulatory activity of IR700-labelled affibody targeting HER2
Source: Cell Death Dis. 2020 Oct 20;11(10):886. doi: 10.1038/s41419-020-03077-6 (PMC7576828; doi:10.1038/s41419-020-03077-6)
Supplement: Supplementary file 2 — Supplementary Figure Legends [file 41419_2020_3077_MOESM2_ESM.docx]

**SUPPLEMENTARY FIGURE LEGENDS**

**Figure S1.** **A)** Z_HER2:2395_–IR700 conjugate was purified from the excess of dye by gel filtration and recovered with an 85% yield. The efficacy of conjugation was confirmed by protein silver staining and fluorescence imaging. *Left:* Z_HER2:2395_ molecules (1 µg) and Z_HER2:2395_–IR700 conjugate (1 µg) were separated by Tricine SDS-PAGE gel electrophoresis; M – molecular weight marker. *Right:* fluorescence image was acquired at 635 nm excitation and 670 nm emission. Both, confirmed the presence of Z_HER2:2395_-IR700 showing a protein band at a slightly higher molecular weight than the reduced affibody molecule The product band was also associated with a strong fluorescence signal **B)** The ESI-MS of the conjugate ([M + H]^+^ expected: 8887.5, found: 8887.4) in comparison to Z_HER2:2395_ molecules. **C)** HER2 receptor saturation curve of Z_HER2:2395_–IR700 conjugate (red line) as compared to non-specific Z_HER2:2395_–IR700 binding in the presence of a 50-fold molar excess of the free Z_HER2:2395_ molecule (black line) in SKOV-3 cells determined by flow cytometry. Data are presented as mean ± SEM (n=3). Kd - the dissociation constant.

**Figure S2.** **A)** HER2 receptor expression level on the surface of cell membrane in selected cancer cell lines as measured by flow cytometry. The cells were incubated with the anti-HER2-FITC antibody for 1 h at 4°C. **B)** Location and internalization of Z_HER2:2395_–IR700 (1 μM; red) in SKOV-3 cells after 1, 4 or 6 h of incubation at 37°C imaged with a confocal microscope. Hoechst (cell nuclei; blue) and LysoTracker (lysosomes; green) were used as counterstaining. **C)** H&E, Ki67 and HER2 immunostaining of SKOV-3 spheroids (~250 μm) sections 96 h after seeding.

**Figure S3.** **A-C)** Decrease in cell viability as assessed by the CellTiter-Glo® luminescent cell viability assay 24 h post-treatment in 2D cells, following 6 h incubation with the Z_HER2:2395_–IR700 (0.01-1 μM) or IR700 (1 μM) and irradiation with 16 J/cm^2^ light dose, was confirmed to be dose dependent and receptor mediated. (A) SKOV-3 HER2+ve cells, (B) BT-474 HER2+ve cells, (C) MDA-MB-468 HER2-ve cells. **D)** Production of reactive oxygen species (ROS; using DCFDA) and singlet oxygen (^1^O_2_; using SOSGR) in SKOV-3 cells, evaluated 10 min post-treatment: 6 h incubation with or without Z_HER2:2395_–IR700 (1 μM) or IR700 (1 μM) with or without irradiation (16 J/cm^2^) compared to control cells (*) or irradiated only cells (^●^). The results were normalized to the control cells. Data are presented as mean ± SEM (n=3). Statistical difference in comparison to the Z_HER2:2395_–IR700 group determined using ANOVA with Dunnett *post hoc* test. ****p≤0.0001, ***p≤0.001, **p≤0.01, *p<0.05. **E)** Inhibition of cell death by pre- and post-treatmet incubation with N-acetylcysteine (NAC, 5 mM) or caspase inhibitor (Z-VAD-FMK, 25 µM) assessed by the CellTiter-Glo® luminescent cell viability assay 24 h post-irradiation in 2D SKOV-3 cells. The results were normalized to the control cells. Data are presented as mean ± SEM (n=3). Statistical difference in comparison to the treated groups not incubated with inhibitors determined using Mann–Whitney t test. ****p≤0.0001, *p<0.05.

**Figure S4.** **A)** Representative histograms of the SKOV-3 cell counts at 1 or 24 h post-treatment: incubation (1 or 6 h) with or without Z_HER2:2395_–IR700 (0.1 or 1 μM) or IR700 (0.1 or 1 μM) with or without irradiation (8 or 16 J/cm^2^) assessed by flow cytometry. Cells stained simultaneously with propidium iodide (PI) and Annexin V (combined with Alexa Fluor™488). Live cells (Annexin V-/PI-); apoptotic cells (Annexin V+/PI-); necrotic cells (Annexin V+/PI+). **B)** Translocation of calreticulin (CRT) onto the cell membrane of SKOV-3 cells 1 h post-treatment: after 1 h incubation with or without Z_HER2:2395_–IR700 (0.1 μM) with or without irradiation (8 J/cm^2^) compared to control cells, evaluated by flow cytometry. Histograms showed as a function of fluorescent signal (secondary anti-rabbit-AlexaFluor™488). Isotype control - cells incubated only with the secondary antibody. **C)** Densitometric analysis of the optical density of protein bands (HMGB1; HSP90 and HSP70) in cells and cell supernatants (medium) of the SKOV-3 line depending on time after treatment (0.1 μM of Z_HER2:2395_–IR700 + 8 J/cm^2^) compared to light exposed cells (8 J/cm^2^). The results are presented as the ratio of the test protein to the quantitative control (β-actin). Data are presented as mean ± SEM (n=3). Statistical difference in comparison to the control group determined using ANOVA with Dunnett *post hoc* test. ****p≤0.0001, ***p≤0.001, **p≤0.01.

**Figure S5.** **A)** Representative flow cytometric diagrams showing shift in CD86 and HLA-DR expression in co-cultured CD14- DC 48 h post-treatment. **B-C)** Normalized MFI of CD86 and HLA-DR expression on DCs post-treatment. Data are presented as mean ± SEM (n=4-5). The graphs represent data from 5 healthy blood donors. Statistical significance in comparison to the iDC group was determined using ANOVA with Holm-Sidak correction test. **p≤0.01, ***p≤0.001, ****p≤0.0001.

**Figure S6.** **A-B)** Fluorescence intensity and tumour-to-background ratio in the BT-474 tumours over time after Z_HER2:2395_–IR700 (0.5, 3 or 18 µg) i.v. injection, measured in regions of interest (ROI) by Living Image® 4.5.2 software*.* **C)** Comparison of mean fluorescence intensities in subcutaneous BT-474 and SKOV-3 tumours 1 h after administration of Z_HER2:2395_–IR700 (0.5, 3 or 18 µg). **D)** Specific binding of Z_HER2:2395_–IR700 to HER2 receptors. Lateral fluorescent full-body image of mice bearing subcutaneous BT-474 tumours: control (saline) and 1 h after injection of Z_HER2:2395_–IR700 (18 μg). **E)** Organs biodistribution 24 h after i.v. administration of the conjugate (18 µg). Photograph and IR700 fluorescence imaging of dissected organs (Ex=675 nm, Em=720 nm).

**Figure S7.** **A)** Weight measurments of mice subjected to treatment using Z_HER2:2395_–IR700 with irradiation - proof-of-concept in vivo studies. **B)** Kaplan-Meier survival curve. *p<0.05 as assessed by Mantel-Cox test.
